# SELF-REPORTED FATIGUE IN PEOPLE WITH POST-COVID-19: IMPACT ON FUNCTIONING IN DAILY LIFE, AND ASSOCIATED FACTORS – A CROSS-SECTIONAL STUDY

**DOI:** 10.2340/jrm.v56.40811

**Published:** 2024-10-15

**Authors:** Christina BROGÅRDH, Elisabeth EKSTRAND, Agneta MALMGREN FÄNGE, Iben AXEN, Kerstin STIGMAR, Eva EKVALL HANSSON

**Affiliations:** 1 Department of Health Sciences, Lund University, Lund; 2 Department of Neurology, Rehabilitation Medicine, Memory Disorders and Geriatrics, Skåne University Hospital, Lund; 3 Department of Hand Surgery, Skåne University Hospital, Malmö; 4 Intervention and Implementation Research for Worker Health, Institute of Environmental Medicine, Karolinska Institutet, Stockholm; 5 Ear- Nose- and Throat Department, Skåne University Hospital, Sweden

**Keywords:** post-acute COVID-19 syndrome, fatigue, activities of daily living, surveys and questionnaires

## Abstract

**Objective:**

To assess (*i*) the impact of self-reported fatigue on functioning in daily life, and (*ii*) the association with sociodemographics, physical capacity, and work ability among people with post-COVID-19.

**Design:**

A cross-sectional study.

**Subjects:**

Adults reporting post-COVID-19 symptoms for at least 2 months.

**Methods:**

Participants were recruited through social media and responded to an online survey between October 2021 and February 2022 regarding sociodemographics, COVID-19 symptoms, comorbidities, physical and mental fatigue, aerobic capacity, and work ability. Descriptive statistics and logistic regression analyses were used.

**Results:**

A total of 614 participants (88% women, mean age 47 years, on average 13 months of symptoms) were included. A majority (≥ 84%) reported both physical fatigue and mental fatigue, according to the Fatigue Severity Scale and Mental Fatigue Scale. The fatigue impacted motivation, physical functioning, work, family, or social life, and increased sensitivity to stress and concentration difficulties. Among the factors, work ability had the strongest association with both physical fatigue and mental fatigue; odds ratio: 0.650 and 0.473, *p* < 0.001, respectively.

**Conclusion:**

This study found that self-reported fatigue is common among people with post-COVID-19, and negatively impacts functioning in daily life. To achieve a sustainable life and work situation, support and targeted rehabilitation interventions may be important.

## LAY ABSTRACT

Despite self-reported fatigue being common following post-COVID-19, there is limited knowledge of how it impacts functioning on daily life, and which factors are related to fatigue. In this study, 614 adults who had experienced post-COVID-19 symptoms for at least 2 months responded to an online survey with questions on sociodemographic factors, COVID-19 symptoms, comorbidities, fatigue, physical fitness, and work ability. We found that a majority (≥ 84%) of the participants perceived both physical fatigue and mental fatigue. Reduced motivation, impact on physical functioning, work, family, or social life were commonly reported, but also increased sensitivity to stress, problems with mental fatiguability, and concentration. Work ability was the factor most strongly related to both physical fatigue and mental fatigue. To achieve a sustainable daily life and work situation, support and targeted interventions to these people may be important.

So far, around 772 million people worldwide have been infected by COVID-19 (1) and the virus still continues to infect people. Most persons fully recover from COVID-19, but between 7% and 32% experience mid- to long-term symptoms that can fluctuate over time, the so-called post-COVID-19 condition (1–3). The condition occurs in people who have been both non-hospitalized and hospitalized during the acute COVID-19 infection but seems to be more common among intensive care unit treated individuals (3). The post-COVID-19 condition is defined as symptoms that have lasted for at least 2 months following a history of probable or confirmed SARS-CoV-2 infection, that cannot be explained by any other diagnosis (4). The mechanisms behind the condition are not fully understood but might be explained by dysregulation of the immune and autonomic nervous systems, and by viral and inflammatory persistence (5, 6). In recent years the virus has changed and become milder, and vaccinations have also reduced the development of severe post-COVID-19 (7). Nevertheless, the condition may lead to a variety of symptoms, such as muscle weakness, chest pain, difficulties with breathing, anosmia/ageusia, fever, tachycardia, gastrointestinal disorders, cognitive dysfunction, headache, and fatigue (8–10). The symptoms can be disabling and persist for months or years (11, 12).

One of the most disabling symptoms following post-COVID-19 is fatigue (8, 9, 13). Fatigue is often defined as “a subjective lack of physical or mental energy (or both) that is perceived by the individual to interfere with usual or desired activities” (14). Some studies have shown that post-COVID-19 fatigue is particularly common in younger persons (< 60 years) and in non-hospitalized persons (11, 15), whereas other studies (13, 16) have reported that old age, female sex, disease severity in the acute phase of infection, comorbidities, and prior diagnosis of depression/anxiety are potential risk factors for fatigue. In our previous study (9) among 766 community-dwelling people with post-COVID-19 symptoms, a majority of the participants perceived fatigue. We also found that fatigue and reduced aerobic capacity increased the odds of experiencing low life satisfaction (9). However, although it is well known that fatigue could be a disabling problem among persons with post-COVID, few studies have thoroughly investigated how it impacts functioning in daily life. Also, more studies are warranted regarding possible factors associated with post-COVID-19 fatigue. Such knowledge is important to be able to provide adequate rehabilitation and to support people to return to sustainable everyday life and work. Thus, the aim of this study was to assess (*i*) the impact of self-reported fatigue on functioning in daily life, and (*ii*) the association with sociodemographics, physical capacity, and work ability among people with post-COVID-19.

## MATERIALS AND METHODS

### Study design

This study has a cross-sectional design and is part of a larger project on long-term consequences after COVID-19 (Life After Covid, “The LAC project”). Here, only data on self-reported fatigue and associated factors are presented. When reporting the data, the STROBE checklist for cross-sectional studies was followed.

### Recruitment of participants

Participants were recruited through an announcement on social media, posted between 21 October and 13 November 2021. They were invited to participate in the project if they met the following criteria: 18 years or older, able to read and understand Swedish, and having had a suspected or confirmed COVID-19 infection with post-COVID symptoms that had lasted for at least 2 months. Information concerning the project was spread via a Facebook page and directed towards people in the 3 most populated regions of Sweden (i.e., Stockholm, Gothenburg, and Skåne). The link to the project was also posted on Instagram and Twitter and could be shared with people outside these areas. Lund University.se hosted the project’s webpage, which included general information on the project, a participant information sheet, and a link to the survey. The survey was open until 12 February 2022.

### Ethics

The study was approved by the Swedish Ethical Review Authority (Dnr 2020-02776), and the principles of the Declaration of Helsinki were followed. All participants gave consent to participate in the study by clicking on a link that directed them to the online survey.

### Data collection

Data collection was performed using REDCap (Research Electronic Data Capture), which is a secure, web-based application designed to support data capture for research studies (17, 18). Questions and questionnaires that were included in the survey were chosen based on potential symptoms and consequences of post-COVID-19 (19). Sociodemographic factors were collected as well as data on fatigue, aerobic capacity, and work ability.

### Sociodemographic factors

Sociodemographic data included age, sex, family situation, residential community characteristics, level of education, provision, comorbidities, perceived COVID-19 symptoms, and duration of post-COVID-19.

### Questionnaires to assess self-reported fatigue, aerobic capacity and work ability

The Fatigue Severity Scale (FSS) was used to assess physical fatigue and lack of energy (20). It consists of 9 statements regarding perceived impact of fatigue on daily life, with response options ranging from 1 (strongly disagree) to 7 (strongly agree). The total score of the FSS ranges from 1 to 7 (mean of the 9 statements), where a higher score indicates greater severity. FSS has demonstrated adequate validity and reliability in people with various diagnoses (14, 21, 22).

The Mental Fatigue Scale (MFS) (23, 24) was used to assess mental fatigue, which often is characterized by difficulties in performing mentally strenuous tasks for more than short periods, irritability, sensitivity to stress, concentration difficulties, and emotional instability. It includes 15 items with response options ranging from 0 (normal function) to 3 (maximal symptoms). The total score is calculated as the sum of items 1–14, and item 15 provides additional information on daytime variation of symptoms (25). The MFS has been developed to capture mental fatigue for persons with various conditions (25) and has demonstrated high internal consistency (23).

The Rating of Perceived Capacity (RPC) scale (26) was used to assess perceived aerobic capacity. The scale has been shown to be a valid tool to estimate aerobic capacity (26, 27). It is based on metabolic equivalents (METs) that are linked to physical activities on a progressive scale. The most strenuous activity that can be sustained for at least 30 min was rated from 1 (sit) to 20 (elite aerobic training).

The Work Ability Score (WAS) (28) was used to assess participants’ work ability, which is shown to be a valid and reliable question (28, 29). The WAS is based on the perceived current work ability in relation to lifetime best and ranges from 0 to 10 (29).

### Statistical analyses

The statistical analyses were performed with SPSS (Statistical Package for the Social Sciences, version 28.0, IBM Corp, Armonk, NY, USA). Descriptive statistics such as means (standard deviations [SD] and range), frequencies and/or medians (interquartile ranges [IQR]) were used to characterize the participants’ demographics, clinical characteristics, self-reported fatigue, aerobic capacity, and work ability. To promote meaningful interpretation of the fatigue data, item scores for FSS and MFS were categorized into 2 and 3 severity levels, respectively (22). For the FSS, item scores between 1 and 3 represented no or minor problems, whereas scores ≥ 4 represented moderate to severe problems. For the MFS, item scores 0–0.5 were categorized as normal function, 1–1.5 as a problem, and 2–3 as pronounced to maximal symptoms. The proportion of participants with fatigue was determined by the recommended cut-off levels for FSS total score (i.e., ≥ 4) (14) and for MFS total score (i.e., ≥ 10.5) (23).

To investigate factors associated with self-reported fatigue, 2 logistic regression models were built. FSS (i.e., physical fatigue) was the dependent variable in 1 of the models, whereas MFS (i.e., mental fatigue) was the dependent variable in the other (dichotomized according to the cut-off levels). The following independent variables were added in the regression building: age (years), sex (man vs woman), family situation (single vs married/partner), educational level (lower vs higher education), comorbidities (no vs yes), time with post-COVID-19 symptoms (months), aerobic capacity (RPC score), and work ability (WAS score).

The multivariable regression model was built with a generous inclusion criterion (*p* ≤ 0.20) so that no potential variable was excluded in the initial stages. First, the associations with physical or mental fatigue were analysed for 1 variable at the time. The odds ratio (OR) with 95% confidence interval (CI), the explanatory value (Nagelkerke R^2^) and *p*-value were calculated. Second, the variable with the lowest *p*-value (if ≤ 0.20) from the univariable analysis was included in the multivariable model building. Thereafter the other variables were tentatively added, 1 at a time, and the model with 2 independent variables (*p* ≤ 0.20) that had the highest explanatory value was kept. The remaining factors were then added again, 1 at a time, so that, in each step, 1 more variable was added to the model as long as the *p*-value of included variables was ≤ 0.20. In the final model, only significant variables (*p* < 0.05) are presented.

## RESULTS

### Participants

A majority of the participants developed their acute COVID-19 infection during the second wave (autumn and winter 2020–2021) and most of them (85%) had not been in need of hospital care. In Table I, the characteristics of the 614 participants are described. Their mean age was 47 (SD 10) years, a majority were women (88%), highly educated (72%), and working (69%). The mean duration of post-COVID-19 was 13 (SD 5) months, and common symptoms reported were fatigue (81%), joint and muscle pain (47%), anosmia/ageusia (42%), and dyspnoea (40%). Also, 39% stated some kind of comorbidities such as asthma, hypertension, reduced thyroid function, and allergies.

**Table I:** Characteristics of the 614 participants

Variable	Values
Age; mean (SD, range)	47 (10, 18–77)
Sex (*n* = 611)	
Men, % (*n*)	12 (72)
Women, % (*n*)	88 (539)
Family situation	
Single, % (*n*)	21 (131)
Married/cohabiting, %	74 (450)
Partner, not cohabiting, % (*n*)	5 (33)
Educational level	
Primary or secondary school (8–12 years), % (*n*)	28 (169)
Higher education (collage/university), % (*n*)	72 (445)
Provision	
Work, % (*n*)	69 (424)
Student grants, % (*n*)	3 (16)
Sickness benefit, % (*n*)	19 (116)
Other sources of income, % (*n*)	9 (58)
Duration of post COVID-19; mean months (SD, range)	13 (5; 2–25)
Common post COVID-19 symptoms	
Fatigue, % (*n*)	81 (499)
Joint and muscle pain, % (*n*)	47 (286)
Anosmia/ageusia, % (*n*)	42 (256)
Dyspnoea, % (*n*)	40 (243)
Comorbidities; Yes, *n* (%)	39 (242)
Asthma, % (*n*)	28 (68)
Hypertension, % (*n*)	13 (31)
Thyroid dysfunction, % (*n*)	12 (30)
Allergies, % (*n*)	12 (30)
Aerobic capacity; mean RPC score (SD, range)	6 (3, 1–20)
Work ability; mean Work Ability Score score (SD, range)	5 (3, 0–10)

SD: standard deviation; RPC: Rating of Perceived Capacity.

### Self-reported impact of fatigue on functioning in daily life

According to FSS, the proportion of participants with physical fatigue (i.e., a total score ≥ 4) was 87%. In Table II, score distribution (%) and median (IQR) scores for each item on the FSS are presented. More than 90% reported that they were easily fatigued, that their motivation was lower when fatigued, and that fatigue interfered with their physical functioning. As many as 83% of the participants reported that fatigue was among their 3 most disabling symptoms. Approximately 85% perceived that fatigue interfered with work, family, or social life; with carrying out certain duties and responsibilities; that fatigue caused frequent problems; and that it prevented sustained physical functioning. Although many reported that exercise brought on their fatigue, 26% did not. Overall, the median item scores ranged from 5 to 7, and the median (IQR) FSS total score was 6.1 (6.7–4.9).

**Table II:** Score distribution (%) and median scores for individual items, as well as total score of the Fatigue Severity Scale (FSS) among the 614 participants

FSS item	Disagreement (1–3)	Agreement (4–7)	Median score (Q3–Q1)
1. My motivation is lower when I am fatigued (*n* = 612)	8	92	6 (7–5)
2. Exercise brings on my fatigue (*n* = 609)	26	74	5 (7–3)
3. I am easily fatigued (*n* = 610)	8	92	6 (7–5)
4. Fatigue interferes with my physical functioning (*n* = 610)	10	90	7 (7–5)
5. Fatigue causes frequent problems for me (*n* = 610)	14	86	6 (7–4)
6. My fatigue prevents sustained physical functioning (*n* = 607)	14	86	7 (7–5)
7. Fatigue interferes with carrying out certain duties and responsibilities (*n* = 609)	14	86	6 (7–5)
8. Fatigue is among my three most disabling symptoms (*n* = 613)	17	83	7 (7–5)
9. Fatigue interferes with my work, family, or social life (*n* = 610)	13	87	7 (7–5)
Total score (max. 7) (*n* = 614)			6.1 (6.7–4.9)

Note: Items are scored on a 7-graded Likert scale, from 1 “strongly disagree” to 7 “strongly agree”. Response options 1–3 indicate no or minor problem (disagreement) and 4–7 a moderate to severe problem (agreement). Total score ranges from 1 to 7 (mean of the 9 items’ sum score). Fatigue = total score ≥4.

According to MFS, the proportion of participants with mental fatigue (i.e., a total score ≥ 10.5) was 84%. In Table III, score distribution (%) and median (IQR) scores for each item on the MFS are presented. The most pronounced symptoms that many reported (54–68%) were sensitivity to stress, mental fatigability, and fatigue in general. Between 42% and 50% reported pronounced problems with mental recovery and concentration. More than half (53–55%) reported problems with lack of initiative, memory problems, and sensitivity to noise. Perceived problems with emotional stability and sensitivity to light were also common (45–48%), whereas 58% of the participants did not experience a need for increased sleep. Overall, the median item scores ranged between 0.5 and 2.0, and the median (IQR) total MFS score was 18.0 (22.5–12.5).

**Table III:** Score distribution (%) and median scores for individual items, as well as total score of the Mental Fatigue Scale (MFS) among the 614 participants

MFS item	Normal function (0–0.5)	Problem (1–1.5)	Pronounced to maximal symptoms (2–3)	Median score (Q3–Q1)
1. Fatigue in general (*n* = 614)	10	36	54	2.0 (2.0–1.0)
2. Lack of initiative (*n* = 614)	16	55	29	1.5 (2.0–1.0)
3. Mental fatigability (*n* = 614)	10	23	67	2.0 (2.0–1.5)
4. Mental recovery (*n* = 610)	21	29	50	1.5 (2.5–1.0)
5. Concentration difficulties (*n* = 614)	17	35	48	1.5 (2.0–1.0)
6. Memory problems (*n* = 614)	21	54	25	1.0 (2.0–1.0)
7. Slowness of thinking (*n* = 614)	20	40	40	1.5 (2.0–1.0)
8. Sensitivity to stress (*n* = 614)	15	17	68	2.0 (2.5–1.0)
9. Emotional instability (*n* = 614)	37	45	18	1.0 (1.5–0.0)
10. Irritability (*n* = 613)	36	37	27	1.0 (2.0–0.5)
11. Sensitivity to light (*n* = 613)	38	48	14	1.0 (1.5–0.0)
12. Sensitivity to noise (*n* = 614)	23	53	24	1.0 (1.5–1.0)
13. Decreased sleep (*n* = 613)	37	38	25	1.0 (2.0–0.0)
14. Increased sleep (*n* = 610)	58	20	22	0.5 (1.5–0.0)
Total score (max. 42) (*n* = 614)				18 (22.5–12.5)

Note: Individual items are scored on a 7-graded scale ranging from 0–3 (0–0.5 = normal function, 1–1.5 = problem, 2–2.5 = pronounced symptom, 3 = maximal symptom). Total score ranges from 0 to 42. Fatigue = total score ≥ 0.5.

### Factors associated with self-reported fatigue

For FSS (i.e., scores > 4), all the independent variables fulfilled the criteria (*p* < 0.20) for being included in the further multivariable model building (see Table IV). In the final multivariable model, 5 significant variables remained. Of these, work ability had the highest explanatory value (Nagelkerke R^2^ 0.277) to physical fatigue with an OR of 0.650 (95% CI 0.540–5.565, *p* < 0.001). Aerobic capacity added another 0.050 to the total model (cumulative Nagelkerke R^2^ value 0.322), whereas age, sex, and educational level together added 0.03. The total model had a total Nagelkerke R^2^ value of 0.353 (*p* < 0.001) (Table IV).

**Table IV:** Logistic regression analyses of factors associated with physical fatigue according to the Fatigue Severity Scale (dichotomized score ≥4) in 614 persons with post-COVID-19

Variables	Univariable regression analysis		
	*p*-value	Odds ratio (95% CI)	Nagelkerke R^2^
Age (years)	0.147	0.983 (0.960 to 1.006)	0.006
Sex (ref men vs women)	< 0.001	2.959 (1.657 to 5.283)	0.036
Family situation (ref married/partner vs single)	0.017	2.414 (1.173 to 4.965)	0.021
Educational level (ref higher education vs lower education)	0.025	1.997 (1.091 to 3.656)	0.017
Comorbidities (ref no vs yes)	0.007	2.064 (1.221 to 3.489)	0.024
Time with post-COVID-19 symptoms	< 0.001	1.085 (1.034 to 1.138)	0.034
Aerobic capacity (RPC score)	< 0.001	0.735 (0.681 to 0.795)	0.199
Work ability (WAS score)	< 0.001	0.575 (0.499 to 0.663)	0.272
Variables	Final multivariable regression model		
	*p*-value	Odds ratio (95% CI)	Cumulative Nagelkerke R^2^
Work ability (WAS score)	<0.001	0.650 (0.560 to 0.754)	0.272
Aerobic capacity (RPC score)	<0.001	0.850 (0.775 to 0.933)	0.322
Age (years)	0.012	0.962 (0.934 to 0.992)	0.335
Sex (ref men vs women)	0.020	2.366 (1.144 to 4.894)	0.343
Educational level (ref higher education vs lower education)	0.049	2.048 (1.004 to 4.179)	0.353

CI: confidence interval; Nagelkerke R^2^ value = pseudo R^2^ value that tells how well the model explains the dependent variable (from 0 to 1).

For MFS (i.e., scores >10.5), all independent variables except age and educational level fulfilled the criteria (*p* < 0.20) for being included in the further multivariable model building (see Table V). In the final model, 2 significant variables remained. Work ability had the highest explanatory value (Nagelkerke R^2^ 0.398) to mental fatigue with an OR of 0.473 (95% CI 0.397–0.562, *p* < 0.001), whereas sex added another 0.029. The total model had a total Nagelkerke R^2^ value of 0.427 (*p* < 0.001) (see Table V).

**Table V:** Logistic regression analyses of factors associated with mental fatigue according to the Mental Fatigue Scale (dichotomized score ≥10.5) in 614 persons with post-COVID-19

Variables	Univariable regression analysis		
	*p*-value	Odds ratio (95% CI)	Nagelkerke R^2^
Age (years)	0.820	0.998 (0.976 to 1i.019)	0.000
Sex (ref men vs women)	< 0.001	3.342 (1.941 to 5.757)	0.047
Family situation (ref married/partner vs single)	0.008	0.888 (0.336 to 6.971)	0.023
Educational level (ref higher education vs lower education)	0.425	0.203 (0.254 to 0.636)	0.002
Comorbidity (ref no vs yes)	< 0.001	2.445 (1.486 to 4.023)	0.038
Time with post-COVID-19 symptoms	0.007	1.062 (1.016 to 1.109)	0.020
Aerobic capacity (RPC score)	< 0.001	0.769 (0.716 to 0.826)	0.153
Work ability (WAS score)	< 0.001	0.465 (0.392 to 0.551)	0.398
Variables	Final multivariable regression model		
	*p*-value	Odds ratio, (95% CI)	Cumulative Nagelkerke R^2^
Work ability (WAS score)	<0.001	0.473 (0.397 to 0.562)	0.398
Sex (ref men vs women)	<0.001	3.482 (1.739 to 6.974)	0.427

Total model *p*-value < 0.001. CI: confidence interval; Nagelkerke R^2^ value = pseudo R^2^ value that tells how well the model explains the dependent variable (0–1).

## DISCUSSION

In this cross-sectional study among people with post-COVID-19 in Sweden, self-reported impact of fatigue on functioning in daily life and associated factors were assessed. Our main findings were that > 84% of the participants reported both physical and mental fatigue, which had a substantial negative impact on everyday life. Among the various factors, work ability had the strongest association with both physical and mental fatigue.

### Physical fatigue and its impact on functioning in daily life

Almost all participants (87%) reported physical fatigue according to the cut-off score of FSS. This is a high proportion but in line with another study among people with post-COVID-19, in which > 90% (30) reported fatigue as assessed by the FSS. However, in a systematic review (13), including 68 studies, the occurrence of fatigue was lower and ranged between 31% and 64%, 3–6 months post-COVID-19. Possible reasons for the different results might be that different fatigue rating scales were used in the studies, and that the populations differed regarding demographic factors, comorbidities, and severity of COVID-19.

Furthermore, according to FSS, more than four-fifths of our participants reported that physical fatigue was among their 3 most disabling symptoms. Many became easily fatigued and had lower motivation, which interfered with physical functioning, work, social life, and with carrying out duties. Similar findings have been reported in the study by Naik et al. (31), in which FSS was used to investigated fatigue among both hospitalized and non-hospitalized people following COVID-19. In that study (31), fatigue was more common among the non-hospitalized than the hospitalized (79% vs 62%) and the highest FSS mean scores were reported for items 1, 3, 4, and 8 (cf. Table II). These findings are very much in line with our study (see Table II), where most participants were not in need of hospital care at the time of the COVID-19 infection. Another interesting finding in the present study was that around one quarter of the participants reported that exercise did not worsen their fatigue. This is an interesting finding, as it has previously been described that malaise after exertion is common in persons with post-COVID-19 (32). However, recent studies have shown that exercise such as aerobic and resistance training of moderate intensity (33), and individualized strength and endurance training, seem to be safe and beneficial in persons with post-COVID, which could improve maximal exercise capacity, fatigue (33, 34), and quality of life (33). In the future, more research is needed on how exercise should be optimally dosed for people with post-COVID, depending on their remaining symptoms, and also organ damage.

### Mental fatigue and its impact on functioning in daily life

Mental fatigue, according to MFS, was also commonly reported among our participants (> 80%), and primarily manifested as increased sensitivity to stress, mental fatigability, and difficulties with mental recovery and concentration. Somewhat surprisingly, nearly 60% responded that they did not experience a need for increased sleep. This result differs somewhat from another study, where taking a daytime nap was common in the presence of post-COVID-19 fatigue (35). Generally, our result on mental fatigue is somewhat difficult to compare with other post-COVID-19 studies as MFS is a rather new rating scale. However, a recent study from Denmark (36) used MFS to assess mental fatigue among individuals with post-COVID-19 symptoms who have been referred to a rehabilitation unit. More than 75% of their participants suffered from moderate to severe mental fatigue and had an MFS total score of 18.5 points (36), which is in line with our study (median total score 18.0 points). Thus, according to MFS, mental fatigue seems be pronounced in persons with post-COVID-19, even more than, for example, among people that have returned to work after stroke, which is worth noting (22).

### Factors associated with self-reported fatigue

In our multivariable regression analyses, we found that higher perceived work ability (WAS score) decreased the odds of experiencing physical and mental fatigue. Work is shown to be an important component of health (37), and in fact more than two-thirds (69%) of our participants were working despite their problems. Low work ability is shown to be associated with low satisfaction with life among those with post-COVID-19 (9), and therefore it may be important to provide to this population individualized support targeting both individual factors and workplace factors (38).

Furthermore, we also found that being a woman increased the odds of experiencing physical and mental fatigue. Other studies have shown that fatigue is more common among women (13, 39), and that women who suffered from a mild COVID-19 infection have a higher risk of developing persistent mental fatigue (40). It is important to raise awareness of this, and that women affected by persistent fatigue are supported in managing their situation.

Our analyses also revealed that increased aerobic capacity (RPC score) and higher age decreased the odds of experiencing physical fatigue. It has previously been reported that reduced aerobic capacity (i.e., maximal and submaximal physical performance) is common among those with post-COVID-19 (41), and new findings indicate that exercise may reduce fatigue (34). How the training protocol should be designed for best possible effect, however, needs more investigation. Regarding age, Klinkhammer et al. (42) have shown that younger age is associated with higher FSS scores (i.e., more fatigue). This is in line with the results of our study and a possible explanation might be that younger people have many commitments to fulfil during a day, which may increase the stress and fatigue levels.

### Clinical implication

As self-reported fatigue is a common among persons with post-COVID-19, which currently cannot be cured by medications, it may be important to offer these individuals various treatment and rehabilitation interventions. This may include support in how to limit the impact of the remaining symptoms, and how to improve functioning in daily life including work. A recent study has shown that an 8-week online supervised group physical and mental health rehabilitation programme can improve health related quality of life, and reduce depression, fatigue and pain in adults with post-COVID-19 (43). Thus, providing person-centred interventions through multidisciplinary post-Covid clinics may be of importance (44). Although booster doses of vaccine may reduce post-COVID-19 symptoms in the future (2), there are still people who may be in need of outpatient care for a long time (3).

### Strengths and limitations

A strength of the present study is the large study population, and that well-established rating scales were used. FSS has been validated in persons who have had COVID-19 (31), but as FSS and MFS assess different aspects of fatigue (22) both scales were used. MFS is a rather new rating scale and a suitable screening tool for assessing work ability in relation to mental fatigue (25). There are also some limitations that should be noted. The participants were recruited through social media, most were younger, well-educated, women, and had not been hospitalized during their COVID-19 infection, which may question the representativeness of a post-COVID-19 cohort. However, by recruiting participants via social media we reached out to many people, with a variety of long-term symptoms. The over-representativeness of women in the study could be due to the fact that women are more frequently on social media and more often have post-COVID-19 (2, 39, 45).

Furthermore, our participants did not have to prove a test-verified COVID-19 infection to be included in the study or to have a confirmed post COVID-19 condition. It has previously been reported that symptoms do not differ between those who have tested positive for COVID-19 infection and those who still perceive symptoms (11). It would have been interesting to investigate more factors in the present study such as depression, emotional status, and cognitive impairments (42), as mental health issues could occur among people with post COVID-19 (3). However, because our participants responded to many questions in the online survey, we had to limit the data collection to a reasonable amount.

### Conclusion

Self-reported physical and mental fatigue seem to be common among people with post-COVID-19, and have a major negative impact on functioning in daily life. To achieve a sustainable life and work situation, support and targeted rehabilitation interventions may be important for these persons.

## References

[1] World Health Organization. World Health Organization (WHO) Coronavirus (COVID-19) Dashboard. Available from: https://covid19.who.int/

[2] Fang Z, Ahrnsbrak R, Rekito A. Evidence mounts that about 7% of US adults have had long COVID. JAMA 2024; 332: 5–6. 10.1001/jama.2024.11370 38848092

[3] Hedberg P, Granath F, Bruchfeld J, Askling J, Sjöholm D, Fored M, et al. Post COVID-19 condition diagnosis: a population-based cohort study of occurrence, associated factors, and healthcare use by severity of acute infection. J Intern Med 2023; 293: 246–258. 10.1111/joim.13584 36478477 PMC9877994

[4] Soriano JB, Murthy S, Marshall JC, Relan P, Diaz JV, Condition WHOCCDWGoP-C-. A clinical case definition of post-COVID-19 condition by a Delphi consensus. Lancet Infect Dis 2022; 22: e102–e7. 10.1016/S1473-3099(21)00703-9 34951953 PMC8691845

[5] Pierce JD, Shen Q, Cintron SA, Hiebert JB. Post-COVID-19 syndrome. Nurs Res 2022; 71: 164–174. 10.1097/NNR.0000000000000565 34653099

[6] Fedorowski A, Olsen MF, Nikesjo F, Janson C, Bruchfeld J, Lerm M, et al. Cardiorespiratory dysautonomia in post-COVID-19 condition: manifestations, mechanisms and management. J Intern Med 2023; 294: 548–562. 10.1111/joim.13652 37183186

[7] Lamkiewicz K, Esquivel Gomez LR, Kühnert D, Marz M. Genome structure, life cycle, and taxonomy of coronaviruses and the evolution of SARS-CoV-2. In: Domingo E, Schuster P, Elena SF, Perales C, editors. Viral fitness and evolution current topics in microbiology and immunology. 439: New York: Springer; 2023. 10.1007/978-3-031-15640-3_9 36592250

[8] Fernandez-de-Las-Penas C, Pellicer-Valero OJ, Navarro-Pardo E, Palacios-Cena D, Florencio LL, Guijarro C, et al. Symptoms experienced at the acute phase of SARS-CoV-2 infection as risk factor of long-term post-COVID symptoms: the LONG-COVID-EXP-CM multicenter study. Int J Infect Dis 2022; 116: 241–244. 10.1016/j.ijid.2022.01.007 35017102 PMC8743274

[9] Ekstrand E, Brogårdh C, Axen I, Malmgren Fänge A, Stigmar K, Ekvall Hansson E. Perceived consequences of Post-COVID-19 and factors associated with low life satisfaction. Int J Environ Res Public Health 2022; 19: 15309. 10.3390/ijerph192215309 36430026 PMC9690380

[10] Ballering AV, van Zon SKR, Olde Hartman TC, Rosmalen JGM, Lifelines Corona Research I. Persistence of somatic symptoms after COVID-19 in the Netherlands: an observational cohort study. Lancet 2022; 400: 452–461. 10.1016/S0140-6736(22)01214-4 35934007 PMC9352274

[11] Davis HE, Assaf GS, McCorkell L, Wei H, Low RJ, Re’em Y, et al. Characterizing long COVID in an international cohort: 7 months of symptoms and their impact. EClinicalMedicine 2021; 38: 101019. 10.1016/j.eclinm.2021.101019 34308300 PMC8280690

[12] Shen Q, Joyce EE, Ebrahimi OV, Didriksen M, Lovik A, Saevarsdottir KS, et al. COVID-19 illness severity and 2-year prevalence of physical symptoms: an observational study in Iceland, Sweden, Norway and Denmark. Lancet Reg Health Eur 2023; 35: 100756. 10.1016/j.lanepe.2023.100756 38115966 PMC10730314

[13] Ceban F, Ling S, Lui LMW, Lee Y, Gill H, Teopiz KM. Fatigue and cognitive impairment in post-COVID-19 syndrome: a systematic review and meta-analysis. Brain Behav Immun 2022; 101: 93–135. 10.1016/j.bbi.2021.12.020 34973396 PMC8715665

[14] Cumming TB, Packer M, Kramer SF, English C. The prevalence of fatigue after stroke: a systematic review and meta-analysis. Int J Stroke 2016; 11: 968–977. 10.1177/1747493016669861 27703065

[15] Norrefalk JR, Borg K, Bileviciute-Ljungar I. Self-scored impairments in functioning and disability in post-COVID syndrome following mild COVID-19 infection. J Rehabil Med 2021; 53: jrm00239. 10.2340/jrm.v53.188 34643243 PMC8638742

[16] Joli J, Buck P, Zipfel S, Stengel A. Post-COVID-19 fatigue: a systematic review. Front Psychiatry 2022; 13: 947–973. 10.3389/fpsyt.2022.947973 PMC9403611 36032234

[17] Harris PA, Taylor R, Minor BL, Elliott V, Fernandez M, O’Neal L, et al. The REDCap consortium: building an international community of software platform partners. J Biomed Inform 2019; 95: 103208. 10.1016/j.jbi.2019.103208 31078660 PMC7254481

[18] Harris PA, Taylor R, Thielke R, Payne J, Gonzalez N, Conde JG. Research electronic data capture (REDCap): a metadata-driven methodology and workflow process for providing translational research informatics support. J Biomed Inform 2009; 42: 377–381. 10.1016/j.jbi.2008.08.010 18929686 PMC2700030

[19] Wade DT. Rehabilitation after COVID-19: an evidence-based approach. Clin Med (Lond) 2020; 20: 359–365. 10.7861/clinmed.2020-0353 32518105 PMC7385804

[20] Krupp LB, LaRocca NG, Muir-Nash J, Steinberg AD. The fatigue severity scale: application to patients with multiple sclerosis and systemic lupus erythematosus. Arch Neurol 1989; 46: 1121–1123. 10.1001/archneur.1989.00520460115022 2803071

[21] Mattsson M, Moller B, Lundberg I, Gard G, Bostrom C. Reliability and validity of the Fatigue Severity Scale in Swedish for patients with systemic lupus erythematosus. Scand J Rheumatol 2008; 37: 269–277. 10.1080/03009740801914868 18612927

[22] Norlander A, Lindgren I, Pessah-Rasmussen H, Gard G, Brogårdh C. Fatigue in men and women who have returned to work after stroke: assessed with the Fatigue Severity Scale and Mental Fatigue Scale. J Rehabil Med 2021; 9; 53: jrm00227. 10.2340/16501977-2863 34383959 PMC8638741

[23] Johansson B, Ronnback L. Evaluation of the mental fatigue scale and its relation to cognitive and emotional functioning after traumatic brain injury or stroke. Int J Phys Med Rehabil 2014; 2: 1000182. DOI: 10.4172/2329-9096.1000182. 10.4172/2329-9096.1000182.

[24] Johansson B, Starmark A, Berglund P, Rodholm M, Ronnback L. A self-assessment questionnaire for mental fatigue and related symptoms after neurological disorders and injuries. Brain Inj 2010; 24: 2–12. 10.3109/02699050903452961 20001478

[25] Johansson B. Screening method for assessment of work ability for patients suffering from mental fatigue. Front Behav Neurosci 2022: 14: 6: 869377. 10.3389/fnbeh.2022.869377 PMC9237561 35775012

[26] Wisén AG, Farazdaghi RG, Wohlfart B. A novel rating scale to predict maximal exercise capacity. Eur J App Physiol 2002; 87: 350–357. 10.1007/s00421-002-0636-y 12172873

[27] Gjestvang C, Stensrud T, Haakstad LA. How is rating of perceived capacity related to VO2max and what is VO2max at onset of training? BMJ Open Sport Exerc Med 2017; 3: e000232. 10.1136/bmjsem-2017-000232 PMC5731224 29259808

[28] El Fassi M, Bocquet V, Majery N, Lair ML, Couffignal S, Mairiaux P. Work ability assessment in a worker population: comparison and determinants of Work Ability Index and Work Ability score. BMC Public Health 2013; 13: 1–10. 10.1186/1471-2458-13-305 23565883 PMC3637198

[29] Kinnunen U, Nätti J. Work ability score and future work ability as predictors of register-based disability pension and long-term sickness absence: a three-year follow-up study. Scand J Publ Health 2018; 46: 321–330. 10.1177/1403494817745190 29212430

[30] Diem L, Fregolente-Gomes L, Warncke JD, Hammer H, Friedli C, Kamber N, et al. Fatigue in post-COVID-19 syndrome: clinical phenomenology, comorbidities and association with initial course of COVID-19. J Cent Nerv Syst Dis 2022; 24: 14. 10.1177/11795735221102727 PMC9130865 35633835

[31] Naik H, Shao S, Tran KC, Wong AW, Russell JA, Khor E. Evaluating fatigue in patients recovering from COVID-19: validation of the fatigue severity scale and single item screening questions. Health and Quality of Life Outcomes 2022; 20: 170. 10.1186/s12955-022-02082-x 36575437 PMC9792925

[32] Ortelli P, Ferrazzoli D, Sebastianelli L, Engl M, Romanello R, Nardone R, et al. Neuropsychological and neurophysiological correlates of fatigue in post-acute patients with neurological manifestations of COVID-19: insights into a challenging symptom. J Neurol Sci 2021; 15: 420. 10.1016/j.jns.2020.117271 PMC7834526 33359928

[33] Araújo TSB, Barros VRAE, Nunes TXD, Remígio de Aguiar IM, Mastroianni WV, de Souza AFJ, et al. Effects of continuous aerobic training associated with resistance training on maximal and submaximal exercise tolerance, fatigue, and quality of life of patients post-COVID-19. Physiother Res Int 2023; 28: e1972. 10.1002/pri.1972 36088642 PMC9539049

[34] Kogel A, Machatschek M, Scharschmidt R, Wollny C, Lordick F, Ghanem M, et al. Physical exercise as a treatment for persisting symptoms post-COVID infection: review of ongoing studies and prospective randomized controlled training study. Clin Res Cardiol 2023; 112: 1699–1709. 10.1007/s00392-023-02300-6 37698618 PMC10584711

[35] Wensink M, Schaap G, Ten Klooster PM, Doggen JMC, van der Palen J, Vonkeman HE, et al. Physical and mental fatigue in post-COVID syndrome and their associations over time: a small-sample ESM-study to explore fatigue, quality of sleep and behaviours. J Psychosom Res 2023: 164: 111084. 10.1016/j.jpsychores.2022.111084 36436226

[36] Nielsen TB, Leth S, Pedersen M, Harbo HD, Nielsen CV, Laursen CH. Mental fatigue, activities of daily living, sick leave and functional status among patients with long COVID: a cross-sectional study. Int J Environ Res Public Health 2022; 19: 14739. 10.3390/ijerph192214739 36429458 PMC9690484

[37] van der Noordt M, H IJ, Droomers M, Proper KI. Health effects of employment: a systematic review of prospective studies. Occup Environ Med 2014; 71: 730–736. 10.1136/oemed-2013-101891 24556535

[38] Fadyl JK, McPherson KM, Schluter PJ, Turner-Stokes L. Factors contributing to work-ability for injured workers: literature review and comparison with available measures. Disabil Rehabil 2010; 32: 1173–1183. 10.3109/09638281003653302 20170279

[39] Fernandez-de-Las-Penas C, Martin-Guerrero JD, Pellicer-Valero OJ, Navarro-Pardo E, Gomez-Mayordomo V, Cuadrado ML, et al. Female sex is a risk factor associated with long-term post-COVID related-symptoms but not with COVID-19 symptoms: the LONG-COVID-EXP-CM Multicenter Study. J Clin Med 2022; 11: 413. 10.3390/jcm11020413 35054108 PMC8778106

[40] Salvato G, Inglese E, Fazia T, Crottini F, Crotti D, Valentini F. The association between dysnatraemia during hospitalisation and post-COVID-19 mental fatigue. J Clin Med 2023; 12: 3702. 10.3390/jcm12113702 37297898 PMC10253452

[41] Beyer S, Haufe S, Meike D, Scharbau M, Lampe V, Dopfer-Jablonka A, et al. Post-COVID-19 syndrome: physical capacity, fatigue and quality of life. PLoSOne 2023; 18: e0292928. 10.1371/journal.pone.0292928 PMC10593222 37870989

[42] Klinkhammer S, Duits AA, Deckers K, Horn J, Slooter AJC, van Heugten CM, et al. A biopsychosocial approach to persistent post-COVID-19 fatigue and cognitive complaints: results of the Prospective Multicenter NeNeSCo Study. Arch Phys Med Rehabil 2024; 105: 826–834. 10.1016/j.apmr.2023.12.014 38228250

[43] McGregor G, Sandhu H, Bruce J, Sheehan B, McWilliams D, Yeung J, et al. Clinical effectiveness of an online supervised group physical and mental health rehabilitation programme for adults with post-covid-19 condition (REGAIN study): multicentre randomised controlled trial. BMJ 2024; 384: e076506. 10.1136/bmj-2023-076506 38325873 PMC11134408

[44] Arienti C, Lazzarini SG, Andrenelli E, Cordani C, Negrini F, Pollini E, et al. Rehabilitation and COVID-19: systematic review by Cochrane Rehabilitation. Eur J Phys Rehabil Med 2023; 59: 800–818. 10.23736/S1973-9087.23.08331-4 38214047 PMC10792873

[45] Ortona E, Malorni W. Long COVID: to investigate immunological mechanisms and sex/gender related aspects as fundamental steps for tailored therapy. Eur Respir J 2022; 59: 2102245. 10.1183/13993003.02245-2021 34531277 PMC8462012

